# Topical Reviews in *Acta Crystallographica F Structural Biology Communications*

**DOI:** 10.1107/S2053230X21011195

**Published:** 2021-11-01

**Authors:** John R. Helliwell, Janet Newman, Mark J. van Raaij

**Affiliations:** a The University of Manchester, Brunswick Street, Manchester, M13 9PL, United Kingdom; b Collaborative Crystallisation Centre (C3), CSIRO, 343 Royal Parade, Parkville, VIC 3052, Australia; c School of Biotechnology and Biomolecular Sciences, UNSW, Sydney, NSW 2052, Australia; d Departamento de Estructura de Macromoleculas, Centro Nacional de Biotecnologia, Consejo Superior de Investigaciones Cientificas, E-28049, Madrid, Spain

**Keywords:** topical reviews, editorial

## Abstract

Editors of *Acta Cryst. F Structural Biology Communications* discuss their plans for topical reviews.

In this November 2021 issue of *Acta Crystallographica F*, we publish the first of a series of concise *Topical Reviews* on structural biology. The review in this issue discusses the crystal structures of the enzyme hy­droxy­methyl­bilane synthase (also known as porphobilinogen deaminase) and is authored by one of us (Helliwell, 2021 ▸). This article is dedicated to the 50-year anniversary of the Protein Data Bank, the macromolecular structure database to which we are proud contributors and grateful users.

Our idea, originating from John Helliwell, is to publish Topical Reviews, a category which is already described in the Notes for Authors, and to focus each review specifically on a coherent group of structures. We would expect authors to consider a typical group containing 3–20 structures (although there are no strict limits) and the resulting discussion would form a concise, up-to-date and forward-looking review of around 10 000 words. Each review would then be refereed in the usual way and, if accepted, appear prominently in the journal. The PDB holds many possibilities for the subjects of such Topical Reviews – they may be on the same enzyme, structures containing the same ligand, the same not very common co-factor or metal ion, or any kind of logical grouping of a not-too-large number of structures.

Eventually, we would like to publish at least one Topical Review per issue – and we hope that a significant number would be from this initiative (Topical Reviews on other structural biology subjects are of course also welcome!). It occurs to us that not only established scientists or group leaders would be able to write these, but also younger scientists such as postdoctoral fellows or PhD students about to submit their theses.

John Helliwell is the Guest Editor who will drive the commissioning and editing of this type of Topical Review. If you would like our feedback on the subject for a review or have a suggestion for other articles in the journal, please get in touch. Together, we look forward to receiving your ideas and your future submissions.
